# Acute Streptococcal Jaundice

**Published:** 1910-08-13

**Authors:** 


### MEDICINE.

### ACUTE STREPTOCOCCAL JAUNDICE.

There are certain cases of very acute illness associated with rapidly increasing jaundice, and it has recently been shown that streptococcal infection is the primary cause of the disease. In one case, for instance, a woman thirty-six years of age, who had eight days previously been confined at term, was sent into hospital with a diagnosis of scarlet fever. She had been taken ill three days previously with signs of puerperal fever, and the day before admission she developed an acute erythematous rash all over her body and her skin and conjunctivæ became jaundiced. On admission the temperature was 105°.6 F., the pulse rate 160, and the patient was obviously very ill. She was fully conscious, but suffered from hiccough, and there were obvious signs of general peritonitis. The jaundice was well marked, the face and conjunctivæ being of a sulphur-yellow colour. The fæces were colourless and the urine contained bile pigment in abundance. The liver came 1½ inch below the ribs and the spleen was enlarged. A blood culture made during life yielded a pure growth of streptococci, which, when inoculated into a rabbit, produced typical erysipelas. The disease was clearly due to acute streptococcal infection *via* the uterus, with streptococcal septicæmia resulting in jaundice.

In another case a patient who seemed to be in perfect health one Sunday was dead upon the following Thursday. It seemed that she had taken a rather long cycle ride upon the Sunday, and that the same evening she came over poorly and aborted early on the Monday morning. Thinking that this was all that was the matter with her, she sent for no doctor, but by the Tuesday morning both she and her husband became much alarmed because she was developing a yellow colour. Her medical man found that she had a temperature of 103° F., that she was obviously jaundiced and severely ill. The jaundice rapidly increased in severity, the urine became almost black, blood cultures yielded pure growths of streptococci, and the patient died comatose on the Thursday morning. At the post-mortem examination, had there been no knowledge of the history of the case, it would have been very difficult indeed to assign the cause of death. Microscopically there was acute necrosis of the hepatic cells, particularly in the central parts of the lobules, and bacteriological cultures from the heart blood and from other organs yielded streptococci; but to the naked eye there was no very obvious lesion of any of the viscera except for their yellow colour, due to staining by bile. There seemed to be no obvious uterine disease, and in this case it was difficult to say whence the absorption of streptococci had occurred. The patient was taken ill with general symptoms before she aborted, so that it seems quite as likely that the abortion was due to the streptococci as that the streptococcal infection was due to the abortion.

Both these cases were exceedingly acute, and it is perhaps doubtful whether life could have been saved by the use of anti-streptococcal serum; but the importance of remembering that jaundice of this kind may be due to a streptococcal septicæmia is very great, for, provided that a sufficiently early diagnosis can be made by blood cultures, it seems probable that life may sometimes be saved by the use of the serum.

